# Early Identification of Sepsis by Emergency Medical Services: Diagnostic Accuracy of Scoring Systems in a Retrospective Cohort

**DOI:** 10.3390/jcm15020827

**Published:** 2026-01-20

**Authors:** Andrea Kornfehl, David Mickerts, Mario Krammel, David Hauer, Sebastian Schnaubelt

**Affiliations:** 1 Department of Emergency Medicine, Medical University of Vienna, 1090 Vienna, Austria; 2 Intensive Care Unit 13i2, Department of Internal Medicine I, Medical University of Vienna, 1090 Vienna, Austria; 3 Emergency Medical Service Vienna, 1030 Vienna, Austria; mario.krammel@wien.gv.at (M.K.);

**Keywords:** sepsis, infection, prehospital, ambulance, emergency medical service

## Abstract

**Background/Objectives:** Emergency Medical Services (EMSs) frequently provide the first medical contact for sepsis patients, but recognition is challenging. This study thus aimed to determine how often EMSs suspect sepsis and to evaluate the diagnostic accuracy of the quick Sequential Organ Failure Assessment (qSOFA), the Prehospital Early Sepsis Detection (PRESEP) score, and the Modified Early Warning Score (MEWS). **Methods:** A retrospective observational study of all EMS transports to one emergency department during a one-month period in 2023 was conducted. Prehospital vital signs, EMS working diagnoses, and final in-hospital diagnoses were abstracted. Scores were calculated post hoc. The primary outcome was the diagnostic accuracy of the EMSs’ working diagnosis of “suspected sepsis.” Secondary outcomes included the sensitivity, specificity, and area under the receiver operating characteristic curve (AUC) of qSOFA, PRESEP, and MEWS. **Results:** Among 786 EMS encounters, 597 met the inclusion criteria. Twelve patients (2.0%) were ultimately diagnosed with sepsis. EMSs explicitly suspected sepsis in three of them (25.0%; sensitivity 16.7%, specificity 99.8%). Retrospective application of scores yielded markedly higher sensitivity: qSOFA 83.3%, PRESEP 91.7%, and MEWS 83.3%. Specificities were 74.2% for qSOFA, 41.2% for PRESEP, and 77.6% for MEWS. The AUCs were 0.838 for qSOFA, 0.695 for PRESEP, and 0.863 for MEWS, with MEWS significantly outperforming PRESEP (*p* = 0.0215). **Conclusions:** EMS personnel rarely labeled patients with sepsis, recognizing 3 of 12 cases (25%). Retrospective use of scoring systems based on routine vital signs substantially improved diagnostic accuracy, with MEWS performing best overall. Structured screening tools should be prospectively validated and potentially implemented in EMS.

## 1. Introduction

Sepsis is defined as a dysregulated host response to infection leading to life-threatening organ dysfunction. [1] It remains a major global health challenge, with estimates suggesting nearly 50 million cases annually and approximately 11 million deaths worldwide. [2] Timely recognition and treatment are essential to reduce mortality and morbidity, yet diagnostic delays remain common, particularly in the prehospital environment. The effectiveness of treatment is strongly time-dependent, and several studies have demonstrated that earlier initiation of antimicrobial therapy and resuscitation measures can improve survival [3].

EMSs often constitute the very first point of medical contact for patients with sepsis. In many regions, between one quarter and one third of patients who are ultimately diagnosed with sepsis present to EMSs before hospital admission [4,5,6]. This makes the prehospital setting a critical window for recognition and early action. Nevertheless, the diagnostic task is difficult. Sepsis frequently presents with nonspecific symptoms such as fever, altered mental status, or tachypnea, which overlap with numerous other medical conditions. Moreover, EMS personnel must assess patients rapidly, under resource constraints, and often without access to laboratory values or imaging [7,8].

To assist in this challenge, several simple clinical scoring systems have been proposed. The quick Sequential Organ Failure Assessment (qSOFA) score was introduced in 2016 as a bedside tool to rapidly identify patients at high risk of poor outcomes. It requires only three parameters—respiratory rate, systolic blood pressure, and mental status—and was designed for rapid application without laboratory data [1,9]. The Prehospital Early Sepsis Prediction (PRESEP) score was developed specifically for EMSs to improve early recognition of sepsis, incorporating routine prehospital parameters into a weighted scoring system [10]. The Modified Early Warning Score (MEWS), initially developed for in-hospital detection of deterioration, has also been adapted for EMSs and shown promise in small studies [11,12].

Despite these advances, uncertainty remains about how well these tools perform in real-world EMS populations and whether they outperform unaided clinical suspicion [13,14]. Furthermore, data on how often EMS clinicians actually suspect sepsis are sparse [15,16]. Understanding both the frequency of EMS recognition and the comparative performance of screening tools is crucial for guiding training, workflow development, and potential implementation of structured screening in EMS.

While previous data have consistently demonstrated that sepsis is frequently underrecognized in the prehospital setting, limited data are available on how structured scoring systems perform under real-world EMS conditions. In particular, comparative analyses of different prehospital screening tools and their practical diagnostic accuracy remain scarce. Moreover, the potential influence of physician-staffed EMS systems on sepsis recognition has not been sufficiently explored.

The objective of this study was therefore twofold: First, it aimed to quantify how frequently EMSs in Vienna documented “suspected sepsis” as their working diagnosis. Second, the study sought to evaluate the diagnostic performance of qSOFA, PRESEP, and MEWS, when retrospectively applied to routinely collected prehospital data, in identifying patients with a final in-hospital diagnosis of sepsis.

## 2. Materials and Methods

### 2.1. Study Design, Patients, and Data Acquisition

This investigation was designed as a retrospective observational cohort study, and it was conducted at the Department of Emergency Medicine of the Medical University of Vienna, a tertiary care academic hospital with an annual census of approximately 70,000 emergency department (ED) visits. The Vienna EMS system is a two-tier model: all ambulances are staffed with paramedics, and emergency physicians are dispatched to high-acuity cases. EMS documentation is fully electronic, enabling detailed retrospective data abstraction.

In- and exclusion criteria were applied as follows:

- All consecutive EMS transports to the study ED between 1 May and 31 May 2023, were screened, and all adults aged 18 years or older brought to the study ED were eligible for inclusion.
- Exclusion criteria were predefined as incomplete prehospital records preventing score calculation, discharge diagnoses clearly incompatible with infection or sepsis (including acute ischemic stroke [International Classification of Diseases 10th revision, ICD-10 I63], acute myocardial infarction [ICD-10 I21], and cardiac arrest [ICD-10 I46]), interfacility transfers, and cases triaged into non-internal medicine categories. These exclusions were applied to focus the analysis on patients in whom sepsis was a realistic differential diagnosis.

From EMS records, data on patient demographics, comorbidities, vital signs measured on scene (systolic blood pressure, heart rate, respiratory rate, body temperature, peripheral oxygen saturation, and level of consciousness documented by the Alert/Verbal/Pain/Unresponsive AVPU, or Glasgow Coma Scale GCS scale), and the EMS working diagnosis were extracted. The latter included free-text entries and coded options such as “suspected sepsis.” In patients ultimately diagnosed with sepsis, the information was additionally recorded whether EMSs had documented “suspected infection.” In addition, the EMS dispatch priority category was documented for each case. These categories range from O (lowest, non-urgent) to E (highest, most urgent). From hospital records, ED triage information using the Emergency Severity Index (ESI) and final in-hospital diagnoses coded according to ICD-10 were obtained.

Final sepsis diagnosis was based on ICD-10 codes consistent with sepsis and septic shock, in alignment with Sepsis-3 definitions [1] as operationalized in administrative coding. Although coding may not always perfectly reflect clinical diagnosis, this served as the reference standard for consistency.

For each patient, qSOFA, PRESEP, and MEWS were retrospectively calculated based on the first set of prehospital vital signs. qSOFA was scored using its three standard components [9]. PRESEP was computed according to the original published definition [10]. MEWS was calculated using heart rate, systolic blood pressure, respiratory rate, temperature, and AVPU [12]. 

The primary outcome of interest was the diagnostic accuracy of the EMS working diagnosis “suspected sepsis” in identifying patients with a final in-hospital diagnosis of sepsis. Secondary outcomes included the diagnostic performance of qSOFA, PRESEP, and MEWS, expressed as sensitivity, specificity, positive predictive value (PPV), negative predictive value (NPV), and area under the curve (AUC) of receiver operating characteristic (ROC) curves. In addition, exploratory analyses regarding the role of an on-scene emergency physician in sepsis recognition were performed.

The study was conducted in accordance with institutional policies and the Declaration of Helsinki. It was approved by the Ethics Committee of the Medical University of Vienna (no. 1650/2024). All data were analyzed in de-identified form, and the need for informed consent was waived.

### 2.2. Statistical Analysis

Statistical analyses were performed using BlueSky Statistics (version 10.4) and R (version 4.5.2). Continuous variables are reported as median values with interquartile ranges (IQR), whereas categorical variables are expressed as absolute numbers and percentages. For comparisons between groups, we used the Mann–Whitney U test for continuous variables and the chi-squared test or Fisher’s exact test for categorical variables, as appropriate.

To evaluate diagnostic accuracy, 2 × 2 contingency tables were constructed. From these, sensitivity, specificity, PPV, and NPV with exact 95% confidence intervals (Wilson method) were calculated. ROC curves were generated for qSOFA, PRESEP, and MEWS, and AUC values were estimated with corresponding 95% confidence intervals. AUCs were compared using the DeLong test for correlated ROC curves. Optimal thresholds for each score were determined using the Youden index, defined as sensitivity + specificity—1.

Missing data were handled using complete-case analysis. Patients with missing variables necessary for score calculation were excluded from the relevant analyses. Given the exploratory and hypothesis-generating nature of the study, a formal sample size calculation was not performed, and no correction for multiple testing was applied. All significance tests were two-sided, and a *p*-value below 0.05 was considered statistically significant.

## 3. Results

### 3.1. Cohort Characteristics

**Figure 1:**
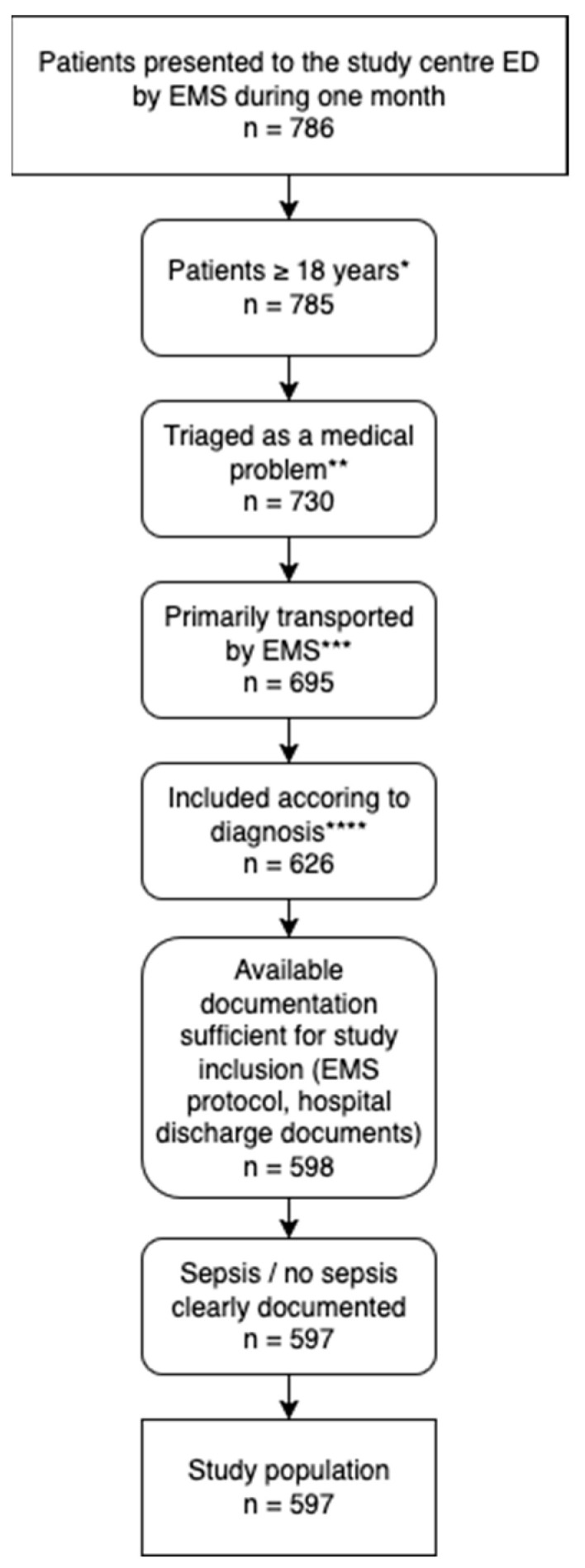
Patient inclusion flowchart with exclusions and final number of cases. ED = Emergency Department; EMS = Emergency Medical Service. * the study center ED only treats adults; ** classified as a problem primarily seen by an internal medicine and emergency medicine specialist and not, for instance, by an ophthalmologist; *** transported as primary EMS-call and not, for instance, as secondary transfer from another hospital; **** excluded if discharge diagnosis not meeting the ICD-10 codes clearly.

During the study period, 786 EMS encounters were recorded. After applying the predefined exclusion criteria, 597 patients remained eligible for analysis (Figure 1). Twelve of these patients (2.0%) had a final in-hospital diagnosis of sepsis. The median age of the study population was 66 years (IQR 50–79), and 46.7% were female. EMS priority categories assigned by the dispatch center were available for all cases. Overall, category C (medium urgency) was most common, accounting for 36.7% of transports, followed by category D (31.0%) and A/B (30.0%). Among sepsis patients, priority C was assigned in half of the cases, while the distribution of other categories was heterogeneous. The most frequent ED triage level was ESI 3 (urgent, but not immediately life-threatening) [17], which applied to 53.1% of all cases. Among patients with final sepsis, however, two-thirds (66.7%) were triaged ESI 1, indicating the highest acuity. Comorbidities were common, with cardiovascular disease and diabetes particularly prevalent. Detailed demographic and clinical characteristics are provided in Table 1 and Table 2.

### 3.2. EMSs’ Suspicion of Sepsis

EMS personnel explicitly documented “suspected sepsis” in only three patients, corresponding to 0.5% of the study cohort. Compared to the 12 patients who were ultimately diagnosed with sepsis, this yielded a sensitivity of 16.7% and a specificity of 99.8%. In addition, four of the 12 sepsis cases were labeled as “suspected infection” but not as sepsis. Taken together, this means that three-quarters of true sepsis cases were at least recognized as infectious in nature, although the specific label of sepsis was rarely applied. The presence of an emergency physician on scene did not significantly increase the likelihood of sepsis recognition (present in 41.7% of sepsis cases vs. 58.3% without physician; *p* = 1.0).

Furthermore, the wording used by EMSs appeared to matter: while “sepsis” was rarely documented, “infection” was noted in half of the actual sepsis cases, suggesting at least partial recognition. Finally, prehospital notification of suspected critical illness (inform the target hospital of the imminent arrival of a potentially instable patient and having an—at least—intermediate care bed ready) strongly influenced subsequent triage: without the notification, 80% of sepsis patients were assigned ESI 2 rather than ESI 1, whereas with it, they were consistently triaged to the highest acuity. The diagnostic accuracy of EMSs’ suspicion is summarized in Table 3.

### 3.3. Performance of Screening Scores

When scores were applied retrospectively to the prehospital data, diagnostic accuracy improved considerably. However, given the limited number of confirmed sepsis cases, analyses of diagnostic accuracy for the scoring systems should be interpreted as exploratory.

- For qSOFA, sensitivity was 83.3% and specificity was 74.2%. The AUC was 0.838 (95% CI 0.709–0.967). The optimal cut-off was ≥1 point, corresponding to a Youden index of 0.575.
- For PRESEP, sensitivity was 91.7% and specificity 41.2%, with an AUC of 0.695 (95% CI 0.546–0.844). The optimal cut-off was ≥1 point (Youden index 0.329). At the originally proposed threshold of ≥4, sensitivity dropped to 41.7% while specificity increased to 83.9%.
- For MEWS, sensitivity was 83.3%, and specificity was 77.6%. The AUC was 0.863 (95% CI 0.777–0.948). The optimal cut-off was ≥3, corresponding to a Youden index of 0.609.

**Figure 2:**
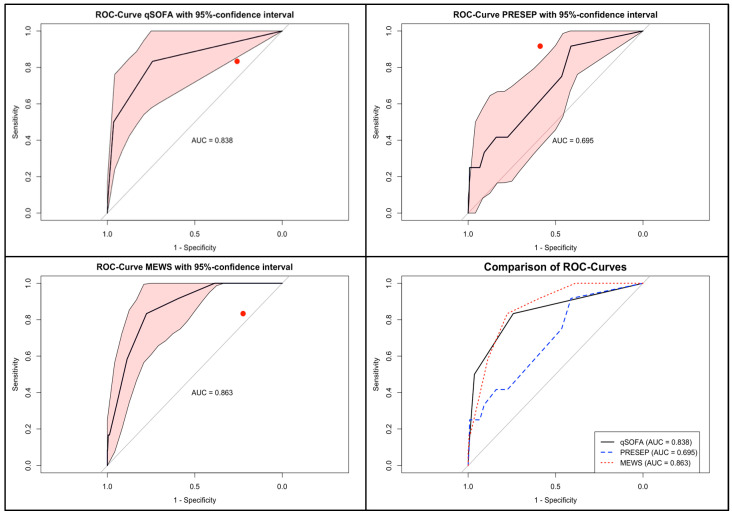
ROC curves for qSOFA, PRESEP, and MEWS with AUCs and 95% confidence intervals.

In direct comparisons of AUCs, MEWS significantly outperformed PRESEP (*p* = 0.0215). Differences between MEWS and qSOFA, and between qSOFA and PRESEP, were not statistically significant. ROC curves are displayed in Figure 2, and detailed results at varying thresholds are presented in Table 4.

## 4. Discussion

This study provides important insights into the recognition of sepsis in the prehospital setting. In the studycohort, EMS personnel in Vienna labeled “suspected sepsis” in only 16.7% of patients ultimately diagnosed with the condition, while 50% of these patients were labeled as having a “suspected infection.” This conservative labeling pattern suggests that EMS clinicians often acknowledge infectious syndromes but hesitate to explicitly classify them as sepsis. Such under-recognition is consistent with previous reports: a Swedish study found that only 36.3% of septic patients were identified prehospitally [5], and a Canadian study reported rates as low as 9% [6]. A systematic review likewise confirmed under-recognition as a persistent challenge [18]. Together, these data highlight the difficulty of sepsis recognition in the field, where heterogeneity of presentation, time pressure, and limited diagnostics contribute to diagnostic uncertainty.

An important additional finding of this study is that the presence of an emergency physician did not significantly increase the likelihood of prehospital sepsis recognition. This observation aligns with previous research showing that even physician-staffed EMS systems rarely outperform paramedic-staffed systems in sepsis recognition [19]. The challenge therefore appears to be more closely related to the unspecific nature of sepsis and the absence of diagnostic resources, rather than the qualification of EMS personnel alone.

Furthermore, the mode of prehospital communication of the finding of (potential) critical illness had a decisive impact on in-hospital triage. When no prehospital alert was issued, the majority of sepsis patients were triaged at ESI level 2, whereas a prealert to the hospital consistently triggered triage at the highest urgency level (ESI 1). This highlights the critical role of EMSs’ communication with receiving hospitals and suggests that structured sepsis alerts could substantially improve early prioritization (e.g., prehospital sepsis alert systems in Portugal which enabled early ED preparedness [20]; studies showing quicker diagnosis and treatment through better integration between EMSs and hospital teams [21]; and improved outcomes associated with structured sepsis alert protocols [22].

The documentation of “infection” versus “sepsis” further illustrates the problem of terminology in the EMS setting. In half of the cases later confirmed as sepsis, EMS providers recorded “suspected infection.” While not equivalent to “suspected sepsis,” this suggests that the infectious nature of the presentation was recognized, but the sepsis label was withheld. This conservative labeling may reflect diagnostic uncertainty or reluctance to assign a term perceived as requiring laboratory or organ dysfunction criteria. It may therefore be pragmatic to consider “suspected infection plus score criteria” as an operational trigger for sepsis alerts in EMSs [18,23,24].

From a screening perspective, retrospective application of three simple scores—qSOFA, PRESEP, and MEWS—to routine prehospital vital signs markedly improved discrimination. In the data at hand, MEWS achieved the highest AUC (0.863), statistically outperforming PRESEP (*p* = 0.0215), and offered the most favorable balance between sensitivity and specificity. qSOFA, though criticized in emergency department populations for low sensitivity, still performed reasonably well in this study’s EMS cohort (AUC = 0.838) [25,26]. PRESEP was highly sensitive but lacked specificity, generating many false positives. These findings align with previous investigations: Bayer et al. reported a sensitivity of 85% and specificity of 86% for PRESEP [10], while Nualprasert et al. described similar performance in a larger cohort [27]. MEWS, originally developed for inpatient monitoring [11], also demonstrated strong discriminatory capacity in prehospital studies, and this study’s results suggest it may be the most balanced option. A broader scoping review further supports the use of structured Early Warning Scores in EMS, even though no score showed optimal performance [24].

The potential value of structured screening is underscored by interventional studies. Implementation of the so-called PRESS protocol increased EMS recognition from 12% to 59% and shortened time to antibiotic administration by 24 min [28]. Similarly, the PHANTASi trial showed that prehospital antibiotics accelerated treatment initiation by around 90 min but did not improve 28-day mortality, likely due to the low incidence of septic shock in the studied population [29]. These mixed results suggest that earlier recognition and notification may improve processes of care, even if outcome effects remain uncertain.

Although formal subgroup analyses were not feasible due to the small number of prehospitally recognized sepsis cases, these patients tended to present with more pronounced physiological abnormalities and higher screening scores, suggesting greater clinical severity. This observation raises the possibility that EMS recognition of sepsis is primarily driven by overt clinical deterioration rather than early or subtle signs. Whether certain findings are overestimated or misinterpreted in the prehospital setting remains unclear and warrants further investigation.

Operational considerations remain crucial. EMSs’ documentation of vital signs is often incomplete; in one large U.S. study, full documentation was present in only 8.2% of encounters, and sepsis suspicion was documented exceedingly rarely [15]. Improved data capture, combined with integration of automated scoring into electronic documentation, could reduce cognitive load and standardize detection. Alerts based on thresholds (e.g., MEWS ≥ 3) may facilitate earlier ED notification and triage. Moreover, the intermediate label “suspected infection” might already serve as a pragmatic step in EMS recognition. Future work should explore whether combining such labeling with structured scoring enhances accuracy and utility. Also, future studies should include larger and multicenter cohorts to validate the diagnostic performance of prehospital screening tools and to allow meaningful subgroup analyses. Prospective investigations evaluating automated score calculation, alert-based thresholds, and real-time clinical decision support may further improve early sepsis recognition. In addition, the impact of structured prehospital screening on downstream clinical outcomes should be assessed.

### Limitations and Strengths

Several limitations must be acknowledged. First, it must be acknowledged that this is a retrospective single-center study. As such, the results should be confirmed in larger, multi-center, prospective studies. Furthermore, the study was conducted in a high-resource metropolitan area, which may limit the applicability of the findings to other settings. Also, the number of patients with confirmed sepsis was small, reflecting the true incidence within the study period and inclusion criteria. This limited sample size restricts statistical power and precludes detailed subgroup analyses. The reference standard relied on ICD-10 coding, which is susceptible to misclassification. Also, scores were calculated retrospectively and may not reflect real-time feasibility or accuracy. Prehospital documentation may be subject to information bias. Further research should also cover other aspects such as the overall patient count with fever or a wider catchment area, as well as potential prior medical contact details.

Despite these limitations, the study has several strengths, including the use of real-world EMS data and head-to-head comparison of three prominent screening tools. Overall, the findings support the hypothesis that structured scoring systems can substantially improve prehospital recognition of sepsis and suggest that MEWS, in particular, represents a promising candidate for implementation.

## 5. Conclusions

EMS personnel documented sepsis in only a small proportion of true cases, underscoring the persistent challenge of early recognition in the prehospital setting. The results demonstrate that the use of simple scoring tools based on routinely collected vital signs substantially improves diagnostic accuracy, with MEWS showing the strongest overall performance. These findings highlight the clinical relevance of structured screening tools and support their potential integration into existing EMS protocols to facilitate earlier sepsis identification. Nevertheless, prospective studies are warranted to validate these findings, assess real-time feasibility, and evaluate the impact of implementing such tools on clinical outcomes.

## Figures and Tables

**Table 1:** Basic characteristics of the study population, including demographics, comorbidities, and vital signs, overall and among patients with and without the final hospital diagnosis of sepsis. EMS = Emergency Medical Service.

	Overall Study Population *n* = 597	Sepsis *n* = 12	No Sepsis *n* = 585
Age, years (IQR)	66 (50–79)	65 (52–74)	66 (50–79)
Female, *n* (%)	279 (47)	7 (58)	272 (47)
Sepsis suspected by EMS, *n* (%)	3 (0.5)	2 (17)	1 (0.2)
*Comorbidities*			
Arterial hypertension, *n* (%)	267 (45)	2 (17)	265 (45)
Diabetes mellitus 2, *n* (%)	130 (22)	1 (8)	129 (22)
Hyperlipidemia, *n* (%)	104 (17)	1 (8)	103 (18)
Coronary disease, *n* (%)	74 (12)	1 (8)	73 (13)
Chronic kidney injury, *n* (%)	58 (10)	2 (17)	56 (10)
Chronic cerebral disease, *n* (%)	35 (6)	1 (8)	34 (6)
Active malignant disease, *n* (%)	46 (8)	5 (42)	41 (7)
Active smoker, *n* (%)	28 (5)	0	28 (5)
Chronic alcohol abuse, *n* (%)	19 (3)	0	19 (3)
*Hospital discharge diagnosis—main medical problem*			
Cardiac, *n* (%)	196 (33)	0	196 (34)
Pulmonary, *n* (%)	99 (17)	2 (17)	97 (17)
Renal, *n* (%)	49 (8)	5 (42)	44 (8)
Gastroenterological, *n* (%)	80 (13)	3 (25)	77 (13)
Infectious, *n* (%)	115 (19)	12 (100)	103 (18)
Upper respiratory tract, *n* (%)	36 (6)	1 (8)	35 (6)
Urogenital, *n* (%)	20 (3)	5 (42)	15 (3)
Oncologic, *n* (%)	23 (4)	1 (8)	22 (4)
Neurologic, *n* (%)	36 (6)	1 (8)	35 (6)
Endocrinologic, *n* (%)	12 (2)	1 (8)	35 (6)
Intoxicative, *n* (%)	25 (4)	0	25 (4)
Other, *n* (%)	74 (12)	0	74 (13)

**Table 2:** Distribution of Emergency Medical Service (EMS) priority categories and Emergency Department (ED) triage levels according to the Emergency Severity Index (ESI), overall and among patients with and without the final hospital diagnosis of sepsis.

	Overall Study Population *n* = 597	Sepsis *n* = 12	No Sepsis *n* = 585
*EMS priority category*			
O, *n* (%)	3 (1)	0	3 (1)
A, *n* (%)	101 (17)	1 (8)	100 (18)
B, *n* (%)	73 (13)	1 (8)	72 (13)
C, *n* (%)	213 (37)	6 (50)	207 (36)
D, *n* (%)	180 (31)	3 (25)	177 (31)
E, *n* (%)	9 (2)	1 (8)	8 (1)
*ED triage level (ESI)*			
1, *n* (%)	95 (16)	8 (73)	87 (15)
2, *n* (%)	165 (28)	3 (27)	162 (28)
3, *n* (%)	313 (53)	0	313 (54)
4, *n* (%)	12 (2)	0	12 (2)
5, *n* (%)	3 (1)	0	3 (1)

**Table 3:** Diagnostic accuracy of Emergency Medical Service (EMS) working diagnosis “suspected sepsis,” including sensitivity (16.7%), specificity (99.8%), positive predictive value (PPV; 66.7%), and negative predictive value (NPV; 98.3%).

	Sepsis	No Sepsis	
Sepsis suspected by EMS	2 (16.7%)	1 (0.2%)	3 → PPV 66.7%
No sepsis suspicion by EMS	10 (83.3%)	584 (99.8%)	594 → NPV 98.3%
	12	585	

**Table 4:** Sensitivity, specificity, positive predictive value (PPV), negative predictive value (NPV), and Youden indices for qSOFA, PRESEP, and MEWS at various thresholds. Within each score, thresholds are listed in descending order of the Youden index. The bottom category “scores” summarizes the optimal cut-offs.

	Sensitivity	Specificity	PPV	NPV	Youden-Index
*qSOFA*					
1	83.3%	74.2%	6.2%	99.5%	0.5752
2	50.0%	96.4%	22.2%	98.9%	0.4641
3	8.3%	99.7%	33.3%	98.1%	0.0799
*PRESEP*					
1	91.7%	41.2%	3.1%	99.6%	0.3286
4	41.7%	83.9%	5.1%	98.6%	0.256
8	25.0%	99.1%	37.5%	98.5%	0.2415
5	33.3%	90.8%	6.9%	98.5%	0.241
7	25.0%	97.9%	20.0%	98.5%	0.2295
*MEWS*					
3	83.3%	77.6%	7.1%	99.6%	0.6094
2	91.7%	59.3%	4.4%	99.7%	0.5098
4	58.3%	88.7%	9.6%	99.0%	0.4705
1	100%	38.6%	3.2%	100%	0.3863
5	33.3%	94.7%	11.4%	98.6%	0.2803
*Score*					
qSOFA ≥ 1	83.3%	74.2%	6.2%	99.5%	0.5752
PRESEP ≥ 1	91.7%	41.2%	3.1%	99.6%	0.3286
MEWS ≥ 3	83.3%	77.6%	7.1%	99.6%	0.6094

## Data Availability

The study data are available from the corresponding author upon reasonable request in accordance with national law and organizational rules.

## Abbreviations

The following abbreviations are used in this manuscript:

AVPU	Alert/Verbal/Pain/Unresponsive
ED	Emergency Department
EMS	Emergency Medical Service
ESI	Emergency Severity Index
GCS	Glasgow Coma Scale
MEWS	Modified Early Warning Score
PRESEP	Prehospital Early Sepsis Detection
qSOFA	Quick Sequential Organ Failure Assessment
